# The Tumor Cell Proliferation Inhibitory Activity of the Human Herpes Virus Type 6 U94 Protein Relies on a Stable Tridimensional Conformation

**DOI:** 10.3390/microorganisms14010255

**Published:** 2026-01-22

**Authors:** Anna Bertelli, Matteo Uggeri, Federica Filippini, Melissa Duheric, Francesca Caccuri, Arnaldo Caruso

**Affiliations:** 1 Section of Microbiology, Department of Molecular and Translational Medicine, University of Brescia, 25123 Brescia, Italyfrancesca.caccuri@unibs.it (F.C.); 2 Lifescience Innovation Good Healthcare Technology—LIGHT s.c.ar.l., 25123 Brescia, Italy

**Keywords:** Human Herpes Virus 6, U94 protein, cancer therapeutics, antiproliferation, *Src* down-regulation, protein modeling, molecular dynamic simulation, artificial intelligence, nucleofection

## Abstract

The U94 protein of Human Herpesvirus 6 exerts antiproliferative effects through downregulation of the *Src* proto-oncogene. We aimed to define the shortest U94 fragment that preserves antiproliferative activity and to explore its structural properties. U94 was truncated into shorter fragments, which were subjected to computational analyses and proliferation assays on MDA-MB-468, BT-549 breast cancer cells. Src phosphorylation levels were scrutinized by Western blot analysis. Data obtained demonstrated that the U94 antiproliferative activity resides in its N-terminal region. Specifically, MT153 (aa 1–153) and MT117 (aa 1–117) fragments exhibited antiproliferative activity, whereas MV85 (aa 1–85) fragment did not. Computational analyses identified MG112 (aa 1–112) and MI108 (aa 1–108) as biologically active and suggested that the β-sheet of the structure is critical. The shortest KI95 fragment (aa 14–108), maintaining a stable β-sheet, demonstrated antiproliferative effects and *Src* downregulation. The antiproliferative activity of U94 and its active fragments relies on stable tridimensional conformation rather than on linear peptide sequence. KI95 represents the shortest active U94 fragment that preserves biological function, with critical residues likely located within the β-sheet region. These findings highlight the importance of structural integrity in U94 functionality and suggest KI95 as a potential therapeutic agent for cancer treatment.

## 1. Introduction

U94 is a highly conserved protein of 490 amino acids (aa) encoded exclusively by the Human Herpes Virus type 6 (HHV-6), an enveloped DNA β-herpesvirus [1,2]. Two variants, HHV-6A and HHV-6B, of this viral strain have been identified, with HHV-6B being responsible for roseola infantum, also known as the sixth disease or exanthema subitum, a common childhood infection [3].

Like all herpesviruses, HHV-6 enters a lifelong latent state in Peripheral Blood Mononucleate Cells (PBMCs) and in some somatic cells following the lytic phase of primary infection, with the ability to reactivate during periods of immunosuppression [4,5]. In this phase, the U94 gene is one of the few expressed. It accumulates in the nucleus, silencing viral replication and modulating the proliferation of latently infected cells, thus suggesting its crucial role for the maintenance of viral latency [6,7]. In vitro co-immunoprecipitation and pull-down assays, as well as in vivo mammalian two-hybrid assays, also showed that the U94 protein interacts with the human TATA-binding protein (hTBP), a fundamental transcription factor belonging to the Pol II-specific complex (TFIID), essential for transcription initiation. Particularly, only the N-terminal portion of U94, from aa 1 to 244, was found to be necessary for binding hTBP, with the region around aa 114 hypothesized to define the boundary [8]. More recently, the U94 protein was found to prevent clonogenicity, tumor growth, and metastasis of aggressive human breast cancer (MDA-MB-231) and human cervical cancer (HeLa) cells [1,9]. These effects were attributed to downregulation of the *Src* proto-oncogene and its downstream signaling pathways. Indeed, the Src protein, a member of the Src family kinases (SFKs), is known to trigger several oncogenic signaling cascades, including JAK-STAT3 (Janus kinase—Signal Transducer and Activator of Transcription 3), Ras-MAPK (Rat sarcoma virus—Mitogen Activated Protein Kinase), PI3K-AKT (Phosphatidylinositol 3-Kinase—alpha serine/threonine-protein kinase), and FAK (Focal Adhesion Kinase)/Paxillin pathways, which are involved in cancer cell proliferation, migration, adhesion, survival, and metastasis [10,11]. Given its crucial role in tumor development and progression, the down-modulation of Src protein by U94 represents a promising strategy to counteract tumor cell growth. Additionally, in vivo assays on NOD/SCID (Non-Obese Diabetic/Severe Combined Immune Deficiency) mice inoculated with U94-expressing MDA-MB-231 cells further highlighted the U94 antiproliferative activity. In fact, a remarkable reduction and delay in tumor growth and metastasis were observed compared to the controls, together with a partial Mesenchymal-to-Epithelial Transition (MET) [9]. Moreover, the U94 antiproliferative properties were also proved in vitro on U87 glioma cells and further confirmed in vivo, where tumor growth was suppressed in nude mice injected with U94-expressing U87 cells [12].

In light of the promising U94 anticancer properties, the present study was aimed to identify the functional epitope responsible for the antiproliferative activity of the viral protein. Through a combined in silico and in vitro approach, here, we show that the *Src* proto-oncogene down-modulation and the consequent antiproliferative activity of the U94 protein do not rely on a short linear aa sequence, but rather on a stable three-dimensional structure.

## 2. Materials and Methods

### 2.1. Cell Lines: MDA-MB-468 and BT-549

Human breast cancer cells (MDA-MB-468 and BT-549) were obtained from the American Type Culture Collection and grown according to ATCC product sheet recommendations.

### 2.2. pVAX Vectors Construction

The U94 fragments were obtained by PCR (Polymerase Chain Reaction) amplification of shorter portions of the HHV-6 U94 gene with specific primers (Table S1) with GoTaq^®^ Green Master Mix (Promega, Singapore) in a 25 μL reaction containing 12.5 μL of GoTaq^®^ Green Master mix, 0.5 μM of sense and antisense primers, 250 ng of template, and nuclease-free water to a final volume of 25 μL. The amplification conditions were as follows: 95 °C for 2 min for the initial denaturation step, followed by 40 cycles (95 °C for 30 s, annealing temperature according to the employed primers for 1 min, 72 °C for an appropriate amount of seconds on the basis of the PCR product length, considering a GoTaq^®^ processivity of 1 kilobase per minute) and a final cycle at 72 °C for 10 min.

Afterwards, PCR products were checked on agarose gel and purified through Wizard^®^ SV Gel and PCR Clean-Up System (Promega), according to the manufacturer’s instructions, and quantified using the Qubit DNA HS Assay Kit (Thermo Fisher Scientific, Waltham, MA, USA).

Subsequently, the cloning procedures of the U94 fragments in the pVAX vector (Thermo Fisher Scientific) were performed as follows. The purified PCR products and the pVAX vector were digested with XbaI and ApaI restriction enzymes (New England Biolabs, Ipswich, MA, USA); the enzyme digestion products were evaluated by agarose gel electrophoresis and were purified from it with QIAquick Gel Extraction Kit (QIAGEN, Hilden, Germany), according to the manufacturer’s instructions. The purified digested U94 fragment of interest and the purified digested pVAX vector were ligated in frame through T4 DNA Ligase (New England Biolabs) for 30 min at room temperature, followed by an inactivation step at 65 °C for 10 min. *Escherichia coli* JM109 strain was transformed by thermal shock with each of these newly obtained constructs and the EndoFree^®^ Plasmid Maxi Kit (QIAGEN) was employed for plasmid purification, according to the manufacturer’s instructions. The obtained Maxipreps were quantified using the Qubit DNA HS Assay Kit (Thermo Fisher Scientific) and the NanoDrop^TM^ One/OneC Microvolume UV-Vis Spectrophotometer (Thermo Scientific^TM^). To assess the success of the newly obtained endotoxin-free pVAX Maxipreps, Sanger sequencing was performed with the BigDye terminator v3.1 cycle sequencing kit on SeqStudio Genetic Analyzer (Thermo Fisher Scientific). The derived sequences were analyzed with Geneious software (v. 11.1.5) (Biomatters Ltd., Auckland, New Zealand).

### 2.3. Proliferation Assays and Cells Counting

Passaged two days before nucleofection, 10^6^ MDA-MB-468 and 10^6^ BT-549 cells were transfected through Nucleofector^TM^ Transfection 2b Device (Lonza^TM^, Basel, Switzerland) with Cell Line Nucleofector^®^ Kit V (Lonza^TM^) and Ingenio Electroporation Solution (Mirus, Madison, WI, USA), respectively, with 2 μg of the pVAX construct containing the U94 fragment of interest. Nucleofection efficiency was 75% and cell viability (% PI negative cells) was around 90%. Twenty-four hours after nucleofection, cells were trypsinized, counted using the trypan blue exclusion method, and seeded in a 24-well-plate at the density of 40,000 cells/well for MDA-468 and 20,000 cells/well for BT-549 cell lines. When the negative control reached confluency, approximately 3 or 4 days post-seeding, cells were trypsinized and counted by the trypan blue exclusion method. The pVAX empty vector and pVAX_MV85 constructs were used as negative controls. Each experimental condition was performed in duplicate in each biological assay.

### 2.4. Protein Modeling

Alpha-fold 2 [13] was used to obtain the tridimensional model of the U94 protein (aa 1–490) and of the MT117 (aa 1–117), MG112 (aa 1–112), MI108 (aa 1–108), KI95 (aa 14–108), and MV85 (aa 1–85) fragments. The following parameters were used: pdb100 as template_mode, 48 num_recycles, 16:32 max_relax_iterations, 2000 max_msa, 16 num_seeds, and enable use_dropout. Five models were obtained for the fragments that further underwent Molecular Dynamic (MD) simulations: MT117, MG112, MI108, and KI95, and one was chosen as described below.

### 2.5. Structure Quality Assessment

The model for MDs was chosen considering different parameters: the Z-score by ProSa-web [14,15], and, after a 5 ns MD (see the MD protocol below), evaluating the average potential energy, the Root Mean Square Deviation (RMSD) of the backbone in the last 1 ns, the percentage of Hydrogen bond (H-bond) retained, and by visual inspection (Table S2). The overall more stable model was chosen. For the MT117 fragment, the RMSD calculation was performed without considering the C-tail (aa 109–117), due to its flexibility. The model 3, 4, 2, and 1 were selected for MT117, MG112, MI108, and KI95 fragments, respectively.

### 2.6. Molecular Dynamic Simulation and Trajectory Analysis

Molecular Dynamic (MD) simulations were carried out using Gromacs 2023.1 [16]. The MT117, MG112, MI108, and KI95-modeled fragments were inserted into a cubic box, solvated using the TIP3P water model, and neutralized with Na^+^ and Cl^−^ ions at a physiological concentration of 0.15 mM. The OPLS-AA force field was used. The systems were minimized and then equilibrated in two steps by 200 ps of NVT (constant volume) and 200 ps of NPT (constant pressure) simulations at 300 K. Then, three independent replicas of 500 ns each were performed for every protein fragment. Trajectory analyses were carried out using the Gromacs tools and VMD (version 1.9.3) [17].

### 2.7. Western Blot Analysis

Nucleofected cells were processed as previously described [18]. Equal amounts of total proteins were resolved on a 10% SDS-polyacrylamide gel and then electroblotted onto a PVDF membrane. The blots were incubated overnight at 4 °C with polyclonal antibody to Src pTyr418 (#44-660G, Thermo Scientific) and monoclonal antibody to Src (#36D10, Cell Signaling Technology, Danvers, MA, USA). The antigen–antibody complex was detected by incubation of the membranes for 1 h at room temperature (RT) with peroxidase-conjugated goat anti-rabbit IgG (Cell Signaling Technology) and revealed using the enhanced chemiluminescence (ECL) reagent (Euroclone, Milan, Italy). Images were acquired by G:Box Chemi XX6/XX9 Gel Documentation System (Syngene, Bengaluru, India) and protein expression was determined using ImageJ (version 15.14.45) (NIH).

## 3. Results

### 3.1. The N-Terminal Region of U94 Is Responsible for the Antiproliferative Activity

**Figure 1:**
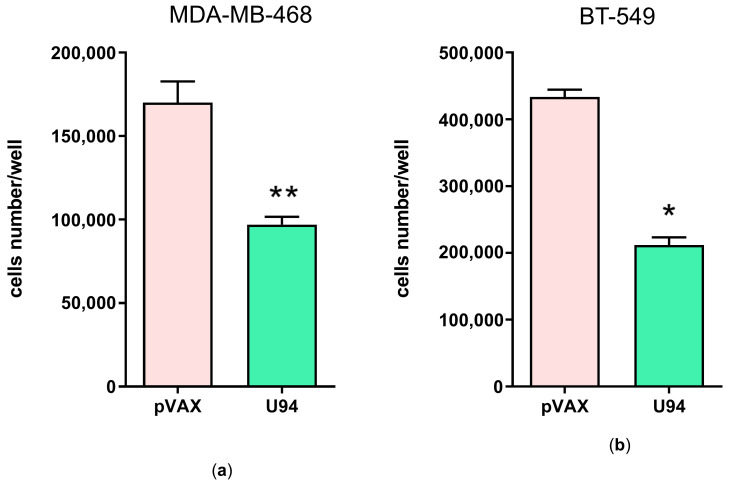
U94 exerts antiproliferative activity on MDA-MB-468 and BT-549 cells. Proliferation assays on (**a**) MDA-MB-468 and (**b**) BT-549 cells, both transfected with pVAX plasmid (negative control) and with the pVAX construct harboring full-length U94 gene. Cells were counted using the trypan blue exclusion method. Bars represent the mean ± SD of two independent experiments performed in duplicate. The statistical analysis was performed by 1-way ANOVA and the Bonferroni post-test was used to compare data (* *p* < 0.05; ** *p* < 0.01 vs. pVAX).

The antiproliferative activity of the U94 protein on the MDA-MB-231 cell line, as well as its ability to impair tumor growth in vivo, has already been demonstrated [9]. Here, we confirmed the U94 antiproliferative activity in vitro on other two human breast cancer cell lines, namely BT-549 and MDA-MB-468. As shown in Figure 1, cells transfected with the pVAX_U94 plasmid and expressing U94 protein showed a decreased growth rate compared to the negative control (pVAX).

**Figure 2:**
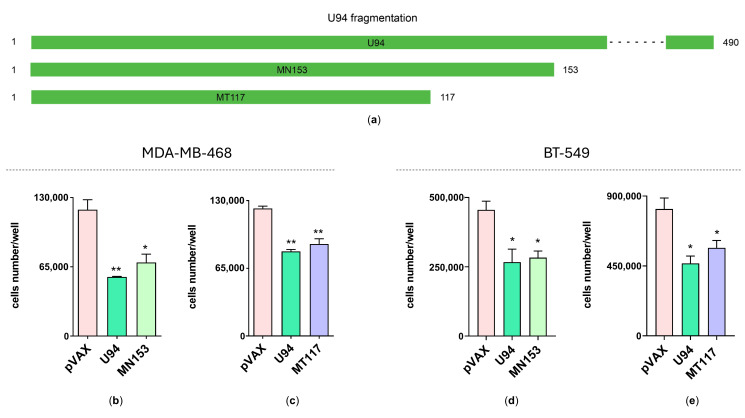
The U94 N-terminal region possesses antiproliferative activity. (**a**) Schematic representation of the U94 fragmentation. The active fragments are shown in green. Proliferation assays on (**b**,**c**) MDA-MB-468 and (**d**,**e**) BT-549 cells. Cells were transfected with pVAX plasmid (negative control) and with pVAX constructs harboring MN153 and MT117 fragments. Cells were counted using the trypan blue exclusion method. Bars represent the mean ± SD of two independent experiments performed in duplicate. The statistical analysis was performed by 1-way ANOVA and the Bonferroni post-test was used to compare data (* *p* < 0.05; ** *p* < 0.01 vs. pVAX).

Mori Y. et al. [8] previously observed the binding between the N-terminal portion (aa 1–244) of U94 and the human TATA-binding protein (hTBP). They also suggested that the coding region around residue 114 may represent the boundary for this interaction. Based on the evidence linking the hTBP overexpression to cell proliferation [19], it can be hypothesized that the antiproliferative activity of U94 is also mediated through its interaction with hTBP. Therefore, the U94 protein was progressively shortened in order to identify its minimal active portion, with a focus on the N-terminal region (Figure 2a). Based on the hydrophobic profile of the U94 N-terminal portion, we initially truncated the U94 gene to generate two fragments composed of 153 aa and 117 aa (MN153; aa 1–153 and MT117; aa 1–117, respectively). Biological data showed that both fragments retained the antiproliferative activity characterizing the full-length protein, significantly decreasing cancer cell growth rate (Figure 2b–e), thus confirming the N-terminal as the active portion of U94.

### 3.2. The U94 Antiproliferative Activity Is Linked to a Three-Dimensional Structure

**Figure 3:**
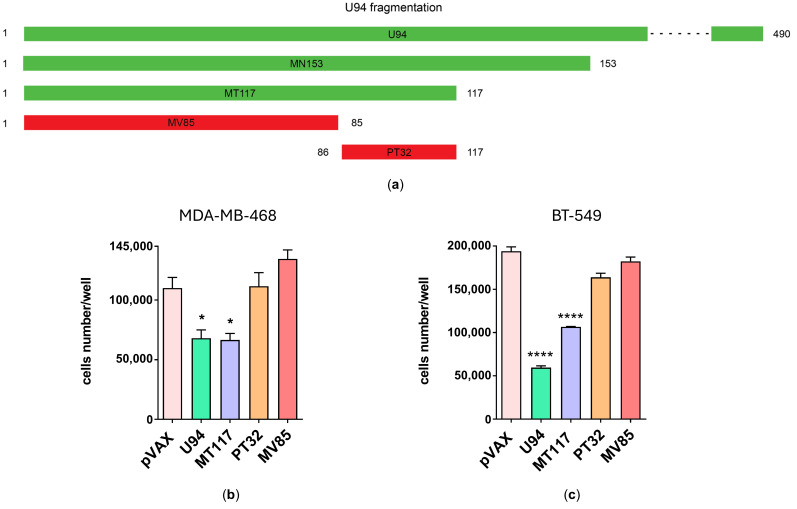
The MV85 and PT32 fragments are inactive in modulating cell proliferation. (**a**) Schematic representation of the U94 fragmentation. In green, the active fragments, while in red, the inactive ones. (**b**,**c**) Proliferation assays on MDA-MB-468 (panel (**b**)) and BT-549 (panel (**c**)) cell lines. Cells were transfected with pVAX plasmid (negative control) and with pVAX constructs harboring U94, MT117, MV85, and PT32 fragments. Cells were counted using the trypan blue exclusion method. Bars represent the mean ± SD of two independent experiments performed in duplicate. The statistical analysis was performed by 1-way ANOVA and the Bonferroni post-test was used to compare data (* *p* < 0.05; **** *p* < 0.0001 vs. pVAX).

To define the minimal U94 sequence retaining antiproliferative activity, the MT117 fragment was further subdivided into two portions: MV85 (aa 1–85) and the PT32 (aa 86–117). As shown in Figure 3b,c, neither the MV85 nor the PT32 fragment displayed antiproliferative activity. In fact, the cancer cells treated with these constructs showed growth rates superimposable to the one of the negative control.

We therefore generated six partially overlapping fragments of the MT117 portion based on its hydrophobic profile to rule out the possibility that the active residues in the MV85 fragment were either inaccessible due to conformational changes or located near the cleavage site at aa 85 (Figure S1a). Interestingly, cancer cell growth was not impaired by any of the newly produced constructs; therefore, they did not exhibit antiproliferative activity (Figure S1b–e). This result suggests that the activity of the U94 protein depends on a specific three-dimensional structure rather than on a linear peptide. Notably, from this point onward, we adopted the pVAX_MV85 construct as the negative control instead of the empty expression vector pVAX, since previous experiments had already demonstrated that both plasmids lacked antiproliferative activity.

**Figure 4:**
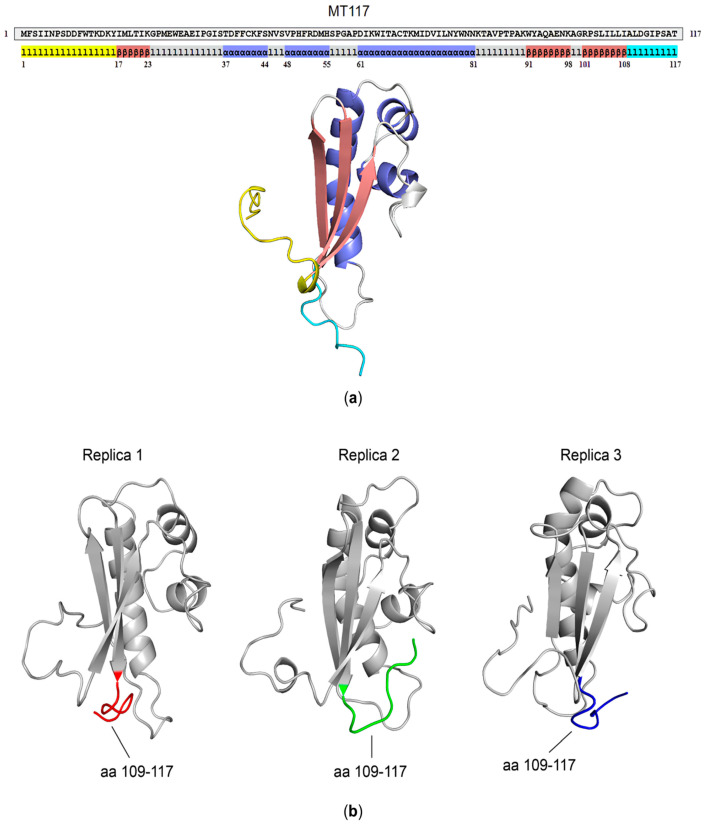
Study of the MT117 structure. (**a**) Three-dimensional model structure of the MT117 fragment. Highlighted in yellow is the N-tail (aa 1–16), in brown the three β-strands (aa 17–23, 91–98 and 101–108) forming the β-sheet, in purple the α-helixes (aa 37–44, 48–55 and 61–81), in azure the C-tail (aa 109–117), and in gray the loops. (**b**) The most representative MT117 protein conformations. Cluster analysis was used to obtain the most represented conformation of the MT117 fragment from replicas 1, 2, and 3. The different spatial positions of the C-tails (aa 109–117) are highlighted in red, green, and blue for replicas 1, 2, and 3, respectively.

To determine whether the maintenance of a proper three-dimensional structure was necessary for preserving biological activity, we computationally analyzed the MT117 protein fragment with the aim of further shortening it. The model structure of the MT117 fragment is characterized by an N-tail, an antiparallel β-sheet composed of three β-strands (β1, β2, and β3), three α-helices, and a C-tail (Figure 4a). While the β1 strand is formed by residues located in the N-terminal portion, the β2 and β3 strands comprise residues located in the C-terminal region of the viral protein. Structural analysis of the MT117 protein suggested that excessive truncation at either terminus could disrupt the β-sheet, potentially compromising the structural integrity and stability of this portion of the protein and, therefore, its biological function.

The MT117 fragment was then subjected to three 500 ns MD replicas. Trajectory analysis highlighted model stability, with a stable RMSD and similar RMSF across the replicas (Figure S2). The C-terminus (aa 109–117) was the most flexible region of the fragment, while the first fifteen residues of the N-terminal portion and the loop formed by aa 82–90 and aa 97–102 also exhibited flexibility in some replicas. However, this did not compromise the overall stability of the protein fragment. Analysis of the most representative conformation from each replica showed that the flexibility of the 82–90 loop was associated with the movement of the C-tail, as their proximity was noticeable across all conformations (Figure S3a). In contrast, in the U94 protein, the 82–90 loop was not expected to exhibit such flexibility due to stabilization by its proximity to the 110–115 loop, which is unable to move freely unlike in the MT117 fragment. Furthermore, U94 has a fourth β-strand (spanning from aa 91 to 98), which interacts with β3, further stabilizing the β-sheet and likely limiting the dynamism of the 82–90 loop (Supplementary Figure S3b). Additionally, the study of the most representative conformation of each replica confirmed the large spatial variability of the C-tail highlighted by the RMSF analysis (Figure 4b).

**Figure 5:**
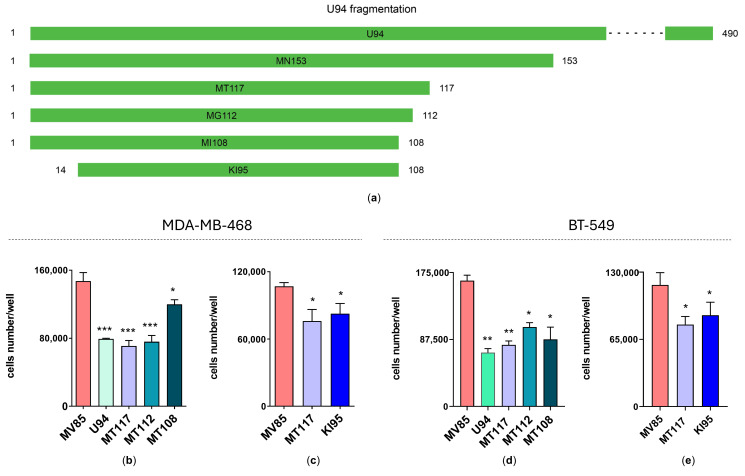
Biological activity of the MT117, MG112, MI108, and KI95 fragments. (**a**) Schematic representation of the active U94-derived fragments in green. (**b**–**e**) Proliferation assays on (**b**,**c**) MDA-MB-468 and (**d**,**e**) BT-549 cells. Cells were transfected with pVAX_MV85 (negative control), with pVAX plasmids harboring U94, MT117, MT112, MT108, and KI95 fragments. Cells were counted using the trypan blue exclusion method. The statistical significance of each condition was calculated compared to MV85 (negative control). Bars represent the mean ± SD of two independent experiments performed in duplicate. Statistical analysis was performed by 1-way ANOVA and the Bonferroni post-test was used to compare data (* *p* < 0.05; ** *p* < 0.01; *** *p* < 0.001 vs. pVAX_MV85).

These observations allowed us to hypothesize that the C-tail is not critical for the conformational stability of the MT117 fragment. Accordingly, a fragment with a shortened C-tail (Figure 5a), namely MG112 (aa 1–112), was modeled and MDs were carried out. Our results confirmed the conformational stability of the newest fragment, characterized by a reduced movement of the C-tail, while the 82–90 loop remained flexible (Figure S4). The computational findings suggested the preservation of the biological activity, which was further confirmed by in vitro experiments. In fact, the MG112 construct efficiently decreased cell growth of both MDA-MB-468 and BT-549 breast cancer cells (Figure 5b,d).

Altogether, these results support the hypothesis that a proper tridimensional conformation is needed to preserve the antiproliferative activity of the U94 protein.

Following this approach, we truncated the C-tail of the MG112 fragment at the end of the β3, generating the MI108 fragment (aa 1–108) (Figure 5a). Trajectory analysis revealed the overall stability of both the β-sheet and the entire fragment (Figure S5). Visual inspection of the most representative conformation of each replica evidenced that β1, formed by residues 17–23 in MT117, was shortened to residues 20–23. However, the number of Hydrogen bond (H-bond) interactions within the β-sheet remained stable compared to MT117 (Figure S6), further supporting its structural stability. As hypothesized based on the computational results, the MI108 construct retained the antiproliferative activity, significantly reducing cancer cell growth of both cancer cell lines in in vitro experiments (Figure 5b,d).

Subsequently, the MI108 fragment was subjected to additional truncation. To preserve the integrity of the β-sheet, further shortening of the C-terminal region was avoided and the attention was directed toward the N-terminal region. Structural analysis of the U94 protein model revealed that the N-terminal portion (aa 1–17) is predicted to be spatially distant from the core structure of the MI108 fragment, with protein folding causing steric hindrance and precluding potential interaction (Figure S7). To assess whether this N-terminal region is critical for the stability and activity of the MI108 fragment, the first 13 aa were removed generating the KI95 fragment (aa 14–108), which was subsequently modeled (Figure 5a). MD results highlighted the overall conformational stability of the fragment (Figure S8). The remaining residues of the N-tail displayed high flexibility, leading to a shortening of β1 similar to that observed in MI108. However, H-bond analysis confirmed the stability of the β-sheet in KI95 (Figure S6). Overall, computational results led us to hypothesize that KI95 retained antiproliferative activity. Indeed, biological assays showed that the KI95 construct significantly diminished the cancer cell proliferation rate to similar levels to those exerted by the MT117 construct (Figure 5c,e). Altogether, our results show that the shortest U94-derived fragment endowed with conformational stability and antiproliferative activity is composed of 95 residues, spanning from aa 14 to 108 of U94 protein.

On the whole, starting from the full-length U94 protein (490 aa), we identified KI95, a 95 aa-long conformationally stable fragment representing the minimal protein sequence retaining the U94 antiproliferative activity.

### 3.3. The U94 Active Residues Are Likely Located Within the β-Sheet

**Figure 6:**
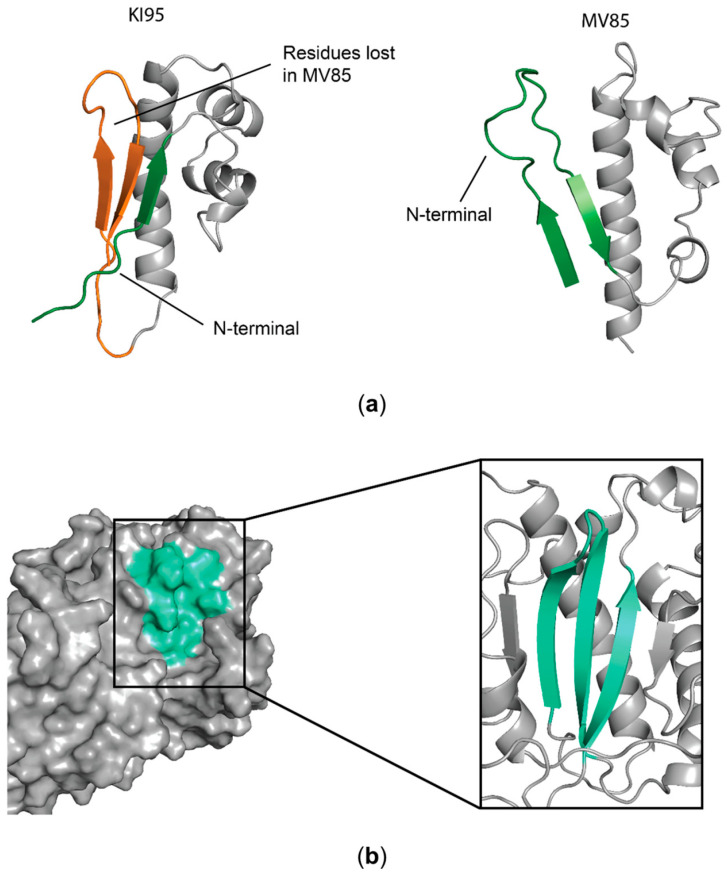
Importance of the KI95 β-sheet. (**a**) Comparison between KI95 (left) and MV85 (right) models. Highlighted in green is the N-terminal portion and in orange the aa residues of KI95 lost in the MV85 fragment; (**b**) in gray is the surface of the U94 protein model. Highlighted in cyan is the exposed β-sheet.

The KI95 fragment was not subjected to further truncation, as any additional shortening could disrupt the β-sheet, resulting in conformational instability and loss of antiproliferative activity. In line with this, structural analysis of the previously characterized inactive MV85 fragment supported our rationale. A structural comparison between the biologically active KI95 and the inactive MV85 fragments revealed a disrupted β-sheet in the latter fragment, coupled with a rearrangement of the N-terminal portion, despite the overall preservation of the α-helix secondary structures and global folding (Figure 6a). Furthermore, in the U94 protein model, the β-sheet under investigation in this study was properly folded and exposed to the solvent, supporting the hypothesis that it plays a critical role in the full-length, non-truncated protein as well (Figure 6b).

These observations suggest that the antiproliferative activity exerted by U94-full length protein and its active fragments may be related to the β-sheet formed by residues 17–23, 91–98, and 101–108, and to its conformational stability.

### 3.4. KI95 Downregulates Src Signaling

**Figure 7:**
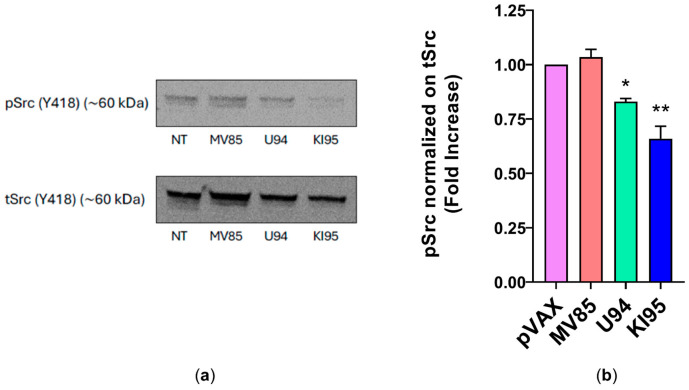
Downregulation of *Src* by U94 protein and KI95 fragment. (**a**) Western blot analysis of MDA-MB-468 lysates. It was performed using antibodies to Src pTyr418 and Src. (**b**) Plot of the p*Src* normalized on tSrc. The quantification was carried out by densitometric analysis. Bars represent the mean ± SD of two independent experiments performed in duplicate. Statistical analysis was performed by 1-way ANOVA and the Bonferroni post-test was used to compare data (* *p* < 0.05; ** *p* < 0.01). NT: not-nucleofected cells.

It has previously been observed that the antiproliferative activity of U94 is associated with downregulation of *Src* [9]. To assess whether the KI95 fragment retains such activity, we performed a Western blot analysis. As shown in Figure 7, 96 h post-nucleofection of MDA-MB-468 cells, expressing either the full-length U94 protein or the KI95 fragment, Src phosphorylation (pY418) was markedly reduced compared to the not-nucleofected cells (NT) or to those nucleofected with the inactive MV85 fragment.

## 4. Discussion

The U94 protein exhibits antiproliferative activity; additionally, its capability to inhibit cell motility, invasion, anchorage-independent growth, and to trigger partial MET was explored both in vitro and in vivo using the cervical cancer cells HeLa and the triple-negative human breast cancer cells (TNBC) MDA-MB-231 [9]. U94 also exerts its anticancer activity on other TNBC cell lines, namely BT-549 and MDA-MB-468, by inhibiting cell cycle [20]. Moreover, U94 ability to inhibit tumoregenesis has also been proved on human glioma U251 and U87, and on prostate cancer cell line PC3 both in vitro and in vivo [12,21,22]. These findings identify the U94 as a promising candidate for cancer treatment, especially considering its ability to selectively inhibit *Src* signaling [9], a key pathway for sustaining tumor progression and metastasis [21].

While the anti-tumor properties of full-length U94 have been extensively studied, focusing on shorter functional fragments represents a critical step for a better understanding of the protein and for potential future applications in vivo. Identifying the minimal active domain of U94 allows for the isolation of its antiproliferative function from other intrinsic roles of the viral protein, thereby facilitating the mapping of the functional interface responsible for this specific biological effect. This characterization is also critical to unlock the design of peptidomimetics or (bio)molecules that can replicate the viral protein activity. In this study, we identified KI95 as the shortest U94 protein portion retaining the antiproliferative activity of U94 on BT-549 and MDA-MB-468 cell lines. However, further studies are needed to investigate KI95 antiproliferative activity on a larger cancer cell line spectrum and in in vivo models.

Specifically, we focused on identifying the U94 functional epitope responsible for its antiproliferative activity. A computational structural approach was employed to strategically truncate the U94 protein, while preserving its biological activity. Computational approaches, such as AI-based molecular modeling and Molecular Dynamic simulations, can be applied to a wide variety of studies [23,24]. One of them is shedding light on protein dynamics, allowing the prediction of protein stability or instability [25,26,27]. Notably, our experiments with shortened peptides derived from U94 protein suggested that the antiproliferative activity characterizing the viral protein is not confined to a linear peptide but relies on three-dimensional structure. Here, computational studies led to the identification of the biologically active 95 aa-long fragment KI95 (aa 14–108). Interestingly, our findings provide an indirect insight into the previously observed interaction between the U94 protein and the hTBP, in relation to the antiproliferative activity of the viral protein. Mori Y. et al. [8] previously suggested that the potential boundary for the U94 interaction with hTBP could be located around the aa 114. Our findings indicate that residues conferring antiproliferative activity to U94 are not located near the aa 114. Our result either suggests that the antiproliferative activity of U94 protein is unrelated to hTBP binding or that the region around residue 114 does not play a significant role in the interaction with hTBP. In both scenarios, further studies are needed to elucidate the biological function of the interaction between U94 and hTBP.

Previous studies [9] showed that the Src family members, known to be involved in tumor development and microenvironment remodeling [10], play a critical role in the U94 antiproliferative activity. In line with this knowledge, here, we show that the shortest U94-derived 95 aa-long active fragment (KI95), endowed with antiproliferative activity, was also able to impair activation of the *Src* signaling pathway in vitro.

From a structural point of view, our findings underscore the complexity of the U94 structure/function relationship. In fact, unlike many proteins from which biologically active linear peptides can be obtained [28], the antiproliferative activity of the U94 protein is strictly dependent on its three-dimensional conformation. Specifically, the comparison between the active KI95 and the inactive MV85 fragments (Figure 3) highlighted the importance of the antiparallel β-sheet formed by three β-strands. Its folding, stability, and exposure to the solvent are likely critical for maintaining the antiproliferative activity since, in the inactive MV85 fragment, the β-sheet is probably disrupted. However, further studies are needed to better define the functional epitope because the current study relies on N- and C-terminal truncations and computational experiments. Even if we have obtained the minimal active protein region (KI95), the contribution of the β-sheet remains to be fully elucidated.

Interestingly, the structural homology between U94 and the Adeno-associated virus type 2 (AAV2) Rep78/68 protein provides important insights into the potential mechanism of the KI95 fragment. Rep78/68 contains a well-defined DNA-binding domain (spanning residues 1–210), homologous and structurally overlapping with the N-terminal region of U94 examined in this study. Cristallography studies of the Rep78/68-DNA complex identified M103, R107, Q111, R138, N139, A141, and G142 as residues that directly bind to the DNA [29]. In the U94 sequence, this region begins at residue 116, downstream of our identified active fragments. Given that fragments MT117 (aa 1–117), MG112 (aa 1–112), MI108 (aa 1–108), and KI95 (aa 14–108) lack this canonical DNA-binding interface, but still exert significant antiproliferative activity and *Src* downregulation, it is likely that direct DNA interaction via this domain does not represent the root cause of the observed activity. This suggests that the KI95 antiproliferative properties may rely on different pathways, such as the interaction with membrane receptors, interference with cell-cycle regulatory proteins, or even the modulation of the tumor microenvironment. While we cannot exclude the presence of a secondary, yet unidentified, DNA-binding motif within the first 108 amino acids of U94, our results underscore that KI95 triggers tumor-suppressive signaling in MDA-MB-468 and BT-549 cell lines.

The link between U94 structural integrity and biological function may become a captivating topic for future research. Site-directed mutagenesis could provide further insights into the antiproliferative activity of the U94 protein, leading to the identification of a small recombinant linear peptide with optimized biological efficacy.

The development of small molecules remains one of the standards for clinical applications. To achieve this goal, identifying the cellular binding partner of the U94 protein is a critical next step. Our data indicate that the viral protein antiproliferative activity relies on a specific 3D conformation, suggesting a structured interface for the interaction with the target(s). Therefore, future studies will prioritize the identification of the host interactome of U94. Affinity purification–mass spectrometry and co-immunoprecipitation represent robust strategies to isolate and identify the specific cellular proteins engaged by the KI95 protein fragment [30,31]. Furthermore, biophysical characterization of these interactions using techniques like surface plasmon resonance will be essential to determine binding kinetics and affinity, thereby validating the target [32,33,34]. Moreover, X-ray crystallography and cryogenic electron microscopy would be valuable methods to shed light on the interaction with the target and to guide drug discovery [35]. Elucidating the interaction interface will unlock the potential of in silico drug discovery. Advanced computational methods can then be leveraged to screen for already approved drugs or small molecules, able to mimic the KI95 pharmacophore, and to obtain the same antiproliferative activity. The integration of artificial intelligence, throughout machine learning or deep learning algorithms, i.e., for the identification of druggable pockets in a protein, the rescoring of docking results, deep-QSAR, ADME, and toxicity prediction, have demonstrated the ability to accelerate the drug discovery process [36,37,38,39,40,41]. This multidisciplinary approach, combining AI-driven computational biology with interactomics, aims to translate the biological activity of U94 into an anticancer therapeutic candidate.

## 5. Conclusions

Here, through a multidisciplinary approach, we demonstrate the antiproliferative activity of the U94-derived shortest fragment KI95 in vitro on two different human breast cancer cell lines. We also associate the U94 biological activity with a proper protein conformational stability by studying its derived fragments, suggesting the role of the identified β-sheet. These findings represent a significant starting point to comprehend the possible future applications and improvements of KI95, presenting a starting point for developing new possible therapeutic approaches.

## Data Availability

The original contributions presented in this study are included in the article/Supplementary Material. Further inquiries can be directed to the corresponding author.

## Supplementary Materials

The following supporting information can be downloaded at: https://www.mdpi.com/article/10.3390/microorganisms14010255/s1, Table S1: List of primers. The forward and reverse primers employed for U94 gene fragmentation are listed below. Table S2: Evaluation of the employed model for 500 ns MD replicas. Values for each model from ProSa-web and analysis of 5 ns MD are reported. The model chosen for further 500ns MD replicas and analyses is underlined in the table. Figure S1: Biological inactivity of shorter fragments. (a) Schematic representation of the U94 fragmentation. In green the active fragments, while in red the inactive ones. (b–e) Proliferation assays on MDA-MB-468 (panels b and c) and BT-549 (panels d–e) cell lines. Cells transfected with pVAX_MV85 (negative control) and with pVAX plasmids harbouring U94, MT117, MI32, TN24, FT29, ID20, WA14 and WT38 fragments. Cells were counted using the trypan blue exclusion method. Bars represent the mean ± SD of two independent experiments performed in duplicate. The statistical significance of each condition was calculated compared to pVAX_MV85 (negative control). Statistical analysis was performed by 1-way ANOVA and the Bonferroni post-test was used to compare data (* *p* < 0.05). Figure S2: Trajectory analyses of the MD replicas of the MT117 fragment. (a) The RMSD of the backbone; (b) The RMSF of the backbone and (c) The superimposition of the most representative conformation of each replica. Figure S3: The flexibility of the 82–90 loop. (a) The proximity of the C-terminal (green) to the 82–90 loop (red) in the most represented conformation of the three replicas. (b) A portion of the U94 protein structure predicted by alpha-fold2. The 82–90 loop (cyan) has less degree of flexibility in U94 due to the spatially constrained 110–115 loop (purple) and the additional β-strand (yellow), which further stabilizes the β-sheet (green), in particular the β2 connected to the 82–90 loop. Figure S4: Trajectory analyses of the MD replicas of the MT112 fragment. (a) The RMSD of the backbone; (b) The RMSF of the backbone and (c) The superimposition of the most representative conformation of each replica. Figure S5: Trajectory analyses of the MD replicas of the MT108 fragment. (a) The RMSD of the backbone; (b) The RMSF of the backbone and (c) The superimposition of the most representative conformation of each replica. Figure S6: β-sheet H-bonds analysis. Comparison in the number of H-bond interactions between the backbone of the residues forming the β-sheet during the MD replicas of the MT117, MI108 and KI95 fragments. Figure S7: U94 3D model. U94 3D model. MI108 protein portion is highlighted in yellow and the N-tail (aa 1–17) in fuchsia. Figure S8: Trajectory analyses of the MD replicas of the KI95 fragment. (a) The RMSD of the backbone; (b) The RMSF of the backbone and (c) The superimposition of the most representative conformation of each replica.

## Abbreviations

The following abbreviations are used in this manuscript:

HHV-6	Human Herpesvirus 6
PBMCs	Peripheral Blood Mononucleate Cells
hTBP	human TATA-binding protein
TFIID	Pol II-specific complex
Ecs	endothelial cells
HeLa	Henrietta Lacks
Src	Rous sarcoma virus tyrosine-protein kinase
SFKs	Src family kinases
JAK-STAT3	Janus Kinase—Signal Transducer and Activator of Transcription 3
Ras-MAPK	Rat sarcoma virus—Mitogen-Activated Protein Kinase
PI3K-AKT	PhosphatIdylinositol 3-Kinase—alpha serine/threonine-protein kinase
FAK	Focal Adhesion Kinase
NOD/SCID	Non-Obese Diabetic/Severe Combined ImmunoDeficiency
PCR	Polymerase Chain Reaction
ATCC	American Type Culture Collection
PVDF	polyvinylidene difluoride
SDS	Sodium Dodecyl Sulfate
MD	Molecular Dynamic
RMSD	Root Mean Square Deviation
H-bond	Hydrogen bond
NVT	constant volume
NPT	constant pressure
RT	room temperature
IgG	immunoglobulin G
ECL	enhanced chemiluminescence reagent
RMSF	Root Mean Square Fluctuation
β1, β2, β3	β-strand 1, 2 and 3
MET	Mesenchymal-to-Epithelial Transition
PPI	Protein–Protein Interaction
SD	standard deviation
*p*	*p*-value
ANOVA	Analysis of Variance
AI	Artificial intelligence
