# Supplementary material for: The Tumor Cell Proliferation Inhibitory Activity of the Human Herpes Virus Type 6 U94 Protein Relies on a Stable Tridimensional Conformation
Source: Microorganisms. 2026 Jan 22;14(1):255. doi: 10.3390/microorganisms14010255 (PMC12844075; doi:10.3390/microorganisms14010255)
Supplement: Supplementary file 1 [file microorganisms-14-00255-s001.zip › microorganisms-4079906-supplementary.pdf]

The tumor cell proliferation inhibitory activity of the Human Herpes Virus type 6 U94 protein relies on a stable tridimensional conformation

Supplementary Materials

Table S1. List of primers. The forward and reverse primers employed for U94 gene fragmentation are listed below.

U94 FRAGMENTS PRIMERS TABLE

U94 fragment		Primers	Amino Acidic Product Size
MN153	Forward	5'-CTTTTCTAGAACCATGTTTTCCATAATAAATCCGAG-3'	153
	Reverse	5’- CTTTGGGCCCTTAATTTTTCTTTGTTTTGGCATACGTGCAC-3’	
MT117	Forward	5'-CTTTTCTAGAACCATGTTTTCCATAATAAATCCGAG-3'	117
	Reverse	5'-CTTTGGGCCCTTACGTTGCGCTGGGAATTCC-3'	
MT108	Forward	5'-CTTTTCTAGAACCATGTTTTCCATAATAAATCCGAG-3'	108
	Reverse	5’-CTTTGGGCCCTTATATCAATAATATTAGGGAGGGTCTG -3’	
MT112	Forward	5'-CTTTTCTAGAACCATGTTTTCCATAATAAATCCGAG-3'	112
	Reverse	5’-CTTTGGGCCCTTATCCATCTAAAGCTATC -3’	
MV85	Forward	5'-CTTTTCTAGAACCATGTTTTCCATAATAAATCCGAG-3'	85
	Reverse	5’- CTTTGGGCCCTTAGACGGCAGTTTTATTATTCC - 3’	
PT32	Forward	5’- CTTTTCTAGAACCATGCCCACCCCTGCAAAGTGG-3’	32
	Reverse	5'-CTTTGGGCCCTTACGTTGCGCTGGGAATTCC-3'	
MI32	Forward	5'-CTTTTCTAGAACCATGTTTTCCATAATAAATCCGAG-3'	32
	Reverse	5’- CTTTGGGCCCTTAGATTTCTGCCTCCCACTC -3’	
TN24	Forward	5’- CTTTTCTAGAACCATGACTATCAAAGGCCCCATG -3’	24
	Reverse	5’- CTTTGGGCCCTTAGTTAGAGAATTTACAG -3’	
FT29	Forward	5’- CTTTTCTAGAACCATGTTCTGTAAATTCTCTAAC-3’	29
	Reverse	5’- CTTTGGGCCCTTAGGTACATGCAGTTATCC -3’	
IV20	Forward	5’- CTTTTCTAGAACCATGATAACTGCATGTACC-3’	20
	Reverse	5’- CTTTGGGCCCTTAGACGGCAGTTTTATTATTCC - 3’	
WA14	Forward	5’- CTTTTCTAGAACCATGTGGAATAATAAAACTGCCG-3’	14
	Reverse	5’-CTTTGGGCCCTTAAGCGTACCACTTTGCAGG-3’	
WT38	Forward	5’- CTTTTCTAGAACCATGTGGAATAATAAAACTGCCG-3’	38
	Reverse	5'-CTTTGGGCCCTTACGTTGCGCTGGGAATTCC-3'	

KI95	Forward	5'-CTTTTCTAGAACCATGAAGGACAAATATATCATG-3'	95
	Reverse	5’-CTTTGGGCCCTTATATCAATAATATTAGGGAGGGTCTG -3’	

Table S2. Evaluation of the employed model for 500 ns MD replicas. Values for each model from ProSa-web and analysis of 5 ns MD are reported. The model chosen for further 500ns MD replicas and analyses is underlined in the table.

MT117				
**Model**	**ProSa-web (Z-score)**	**Epot (^05) Kcal/mol**	**RMSD (Å) (aa 1-108)**	**% H-bond**
1	-4.25	-6.57	1.97	96.55
2	-4.32	-7.70	1.90	98.33
3	-4.66	-5.52	2.40	100.00
4	-4.71	-5.48	3.04	100.00
5	-4.47	-5.51	2.00	98.28
**MG112**				
**Model**	**ProSa-web (Z-score)**	**Epot (^05) Kcal/mol**	**RMSD (Å) (aa 1-108)**	**% H-bond**
1	-4.26	-6.36	2.36	98.44
2	-4.32	-6.55	2.56	100.00
3	-4.03	-6.36	2.91	96.77
4	-4.36	-5.83	2.41	100.00
5	-4.08	-6.54	2.46	98.93
**MI108**				
**Model**	**ProSa-web (Z-score)**	**Epot (^05) Kcal/mol**	**RMSD (Å) (aa 1-108)**	**% H-bond**
1	-4.16	-5.99	3.06	98.21
2	-4.31	-5.87	3.01	100.00
3	-3.99	-6.22	3.91	98.25
4	-4.02	-5.84	2.65	100.00
5	-3.52	-6.03	2.88	96.55
**KI95**				
**Model**	**ProSa-web (Z-score)**	**Epot (^05) Kcal/mol**	**RMSD (Å) (aa 1-108)**	**% H-bond**
1	-4.06	-9.55	2.10	98.25
2	-4.05	-6.25	1.81	100.00
3	-4.32	-6.19	2.34	98.18
4	-4.25	-6.03	1.83	100.00
5	-4.08	-6.40	3.20	100.00

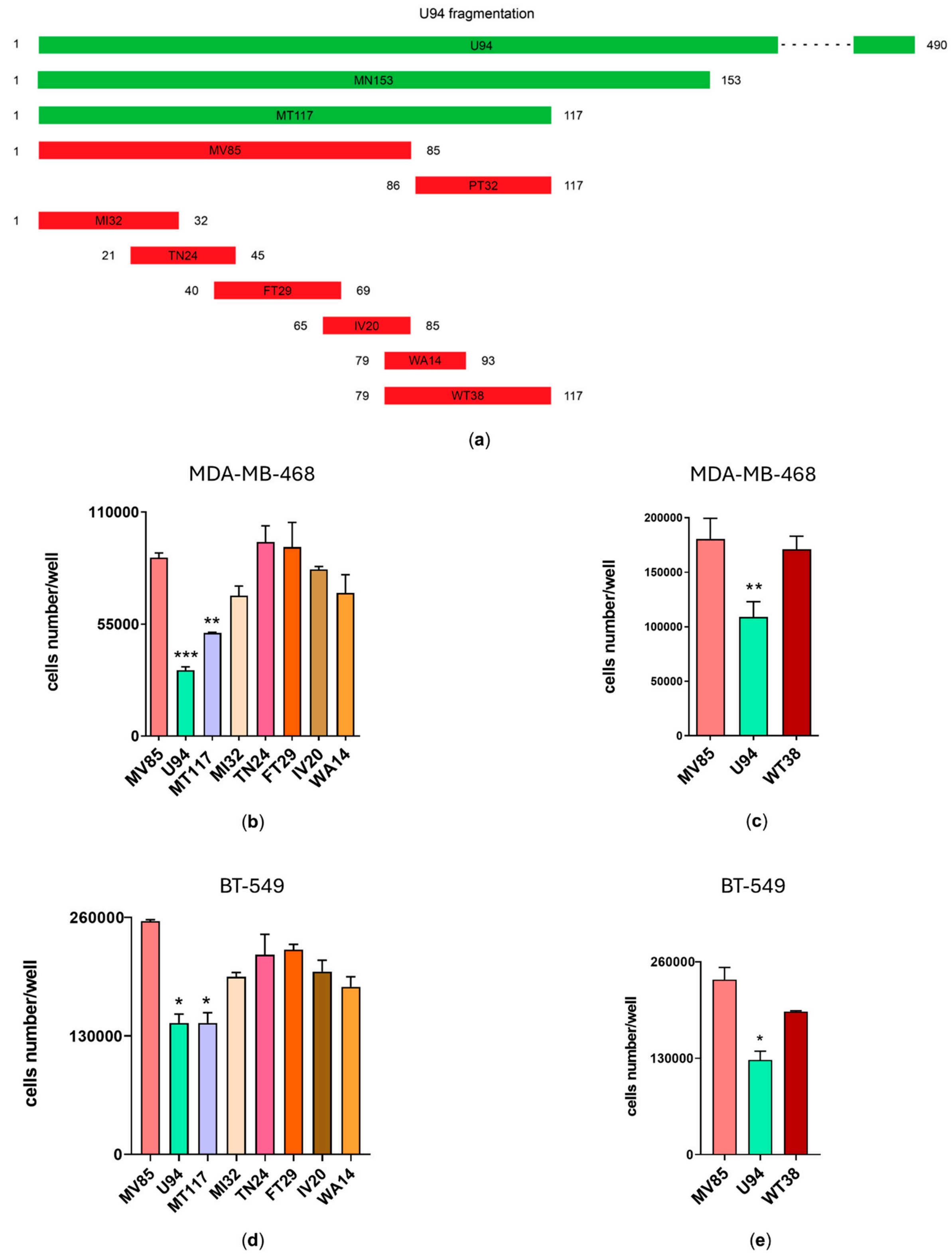

Figure S1. Biological inactivity of shorter fragments of MT117. (**a**) Schematic representation of the U94 fragmentation. In green the active fragments, while in red the inactive ones. (**b-e**) Proliferation assays on MDA-MB-468 (panels **b** and **c**) and BT-549 (panels **d-e**) cell lines. Cells transfected with pVAX_MV85 (negative control) and with pVAX plasmids harbouring U94, MT117, MI32, TN24, FT29, ID20, WA14 and WT38 fragments. Cells were counted using the trypan blue exclusion method. Bars

represent the mean ± SD of two independent experiments performed in duplicate. The statistical significance of each condition was calculated compared to pVAX_MV85 (negative control). Statistical analysis was performed by 1-way ANOVA and the Bonferroni post-test was used to compare data (*P < 0.05).

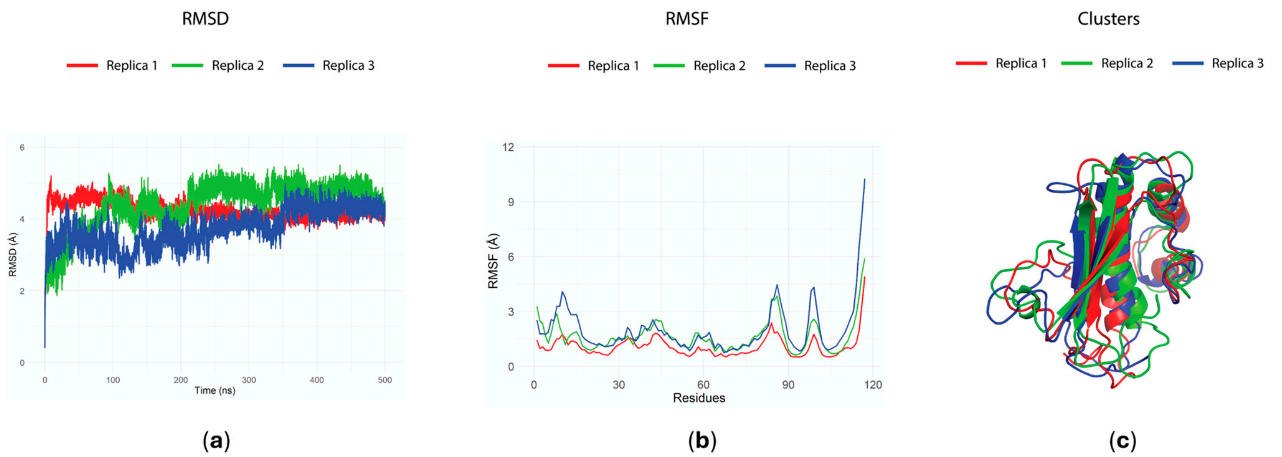

Figure S2. Trajectory analyses of the MD replicas of the MT117 fragment. (**a**) The RMSD of the backbone; (**b**) The RMSF of the backbone and (**c**) The superimposition of the most representative conformation of each replica.

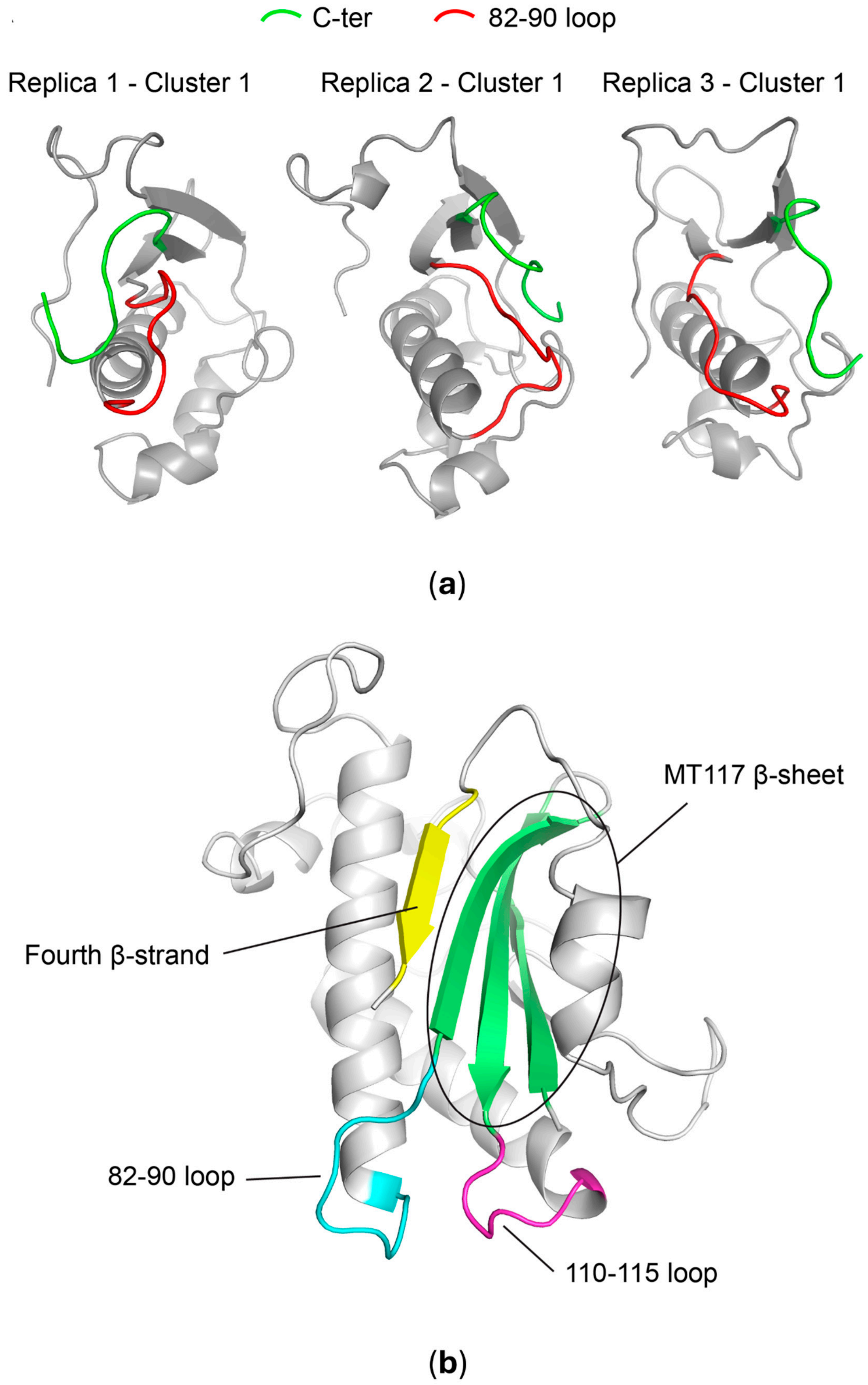
C-ter
82-90 loop
Replica 1 - Cluster 1
Replica 2 - Cluster 1
Replica 3 - Cluster 1
(a)
MT117 β-sheet
Fourth β-strand
82-90 loop
110-115 loop
(b)

Figure S3. The flexibility of the 82-90 loop. (**a**) The proximity of the C-terminal (green) to the 82-90 loop (red) in the most represented conformation of the three replicas. (**b**) A portion of the U94 protein structure predicted by alpha-fold2. The 82-90 loop (cyan) has less degree of flexibility in U94 due to the spatially constrained 110-115 loop (purple) and the additional β-strand (yellow), which further stabilizes the β-sheet (green), in particular the β2 connected to the 82-90 loop.

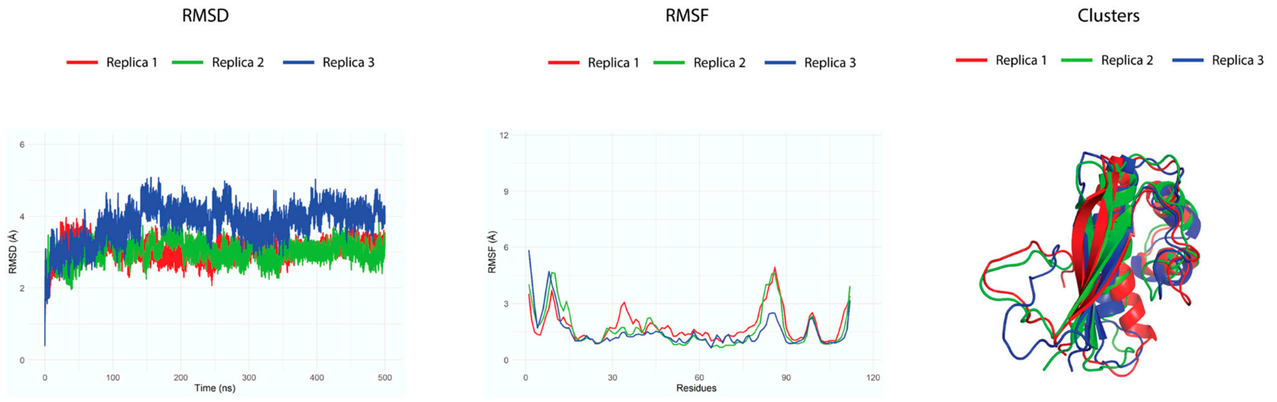

(**a**) (**b**) (**c**)

Figure S4. Trajectory analyses of the MD replicas of the MT112 fragment. (**a**) The RMSD of the backbone; (**b**) The RMSF of the backbone and (**c**) The superimposition of the most representative conformation of each replica.

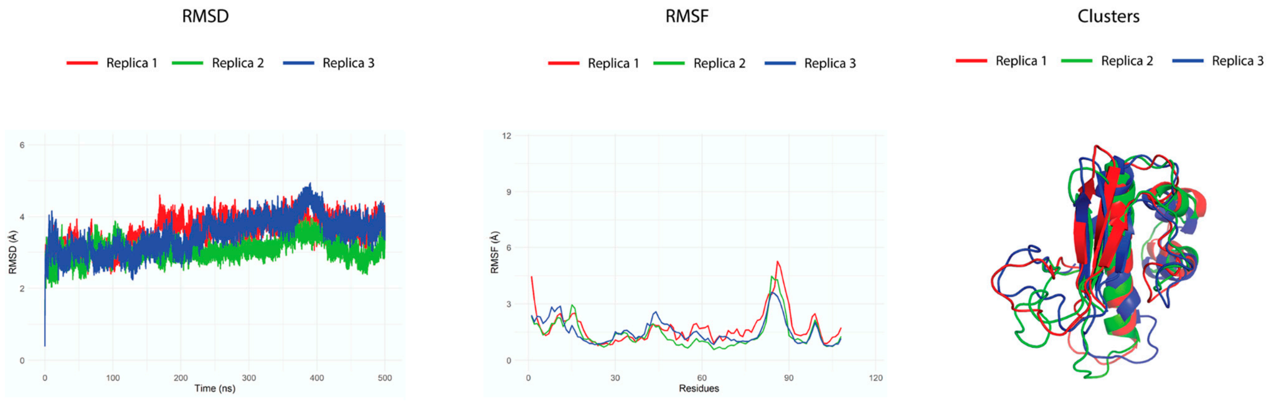

(**a**) (**b**) (**c**)

Figure S5. Trajectory analyses of the MD replicas of the MT108 fragment. (**a**) The RMSD of the backbone; (**b**) The RMSF of the backbone and (**c**) The superimposition of the most representative conformation of each replica.

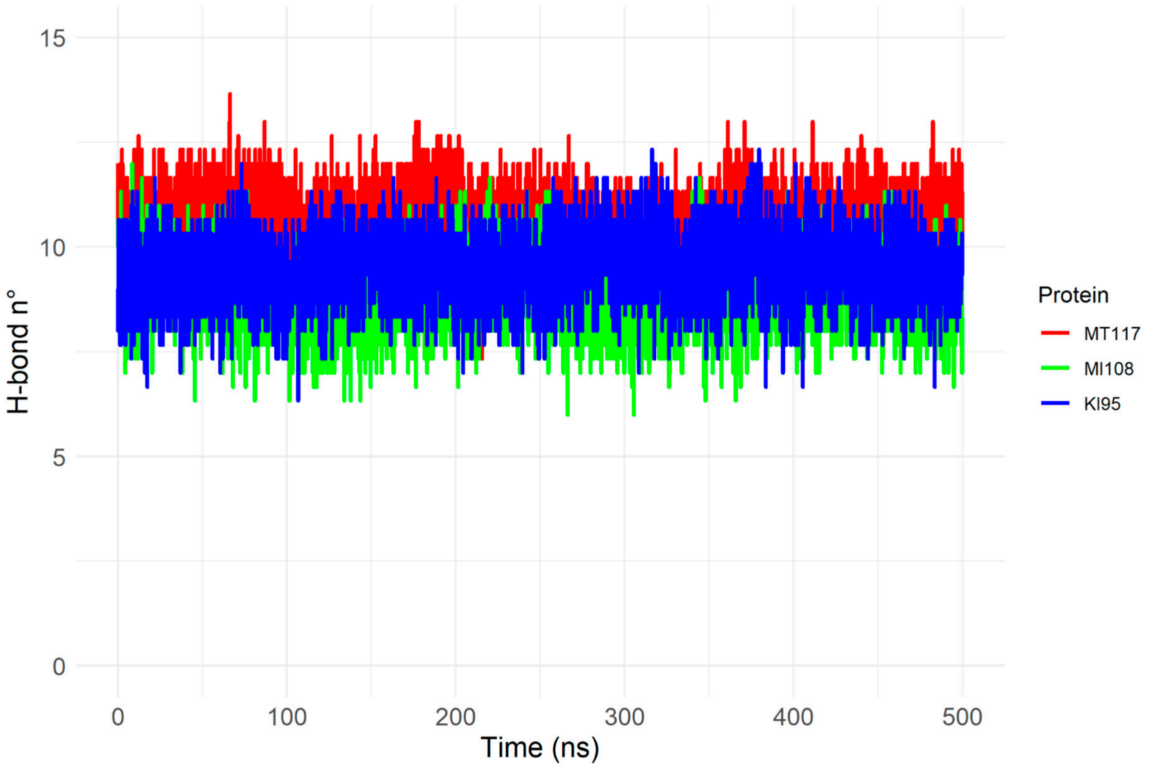

Figure S6. β-sheet H-bonds analysis. Comparison in the number of H-bond interactions between the backbone of the residues forming the β-sheet during the MD replicas of the MT117, MI108 and KI95 fragments.

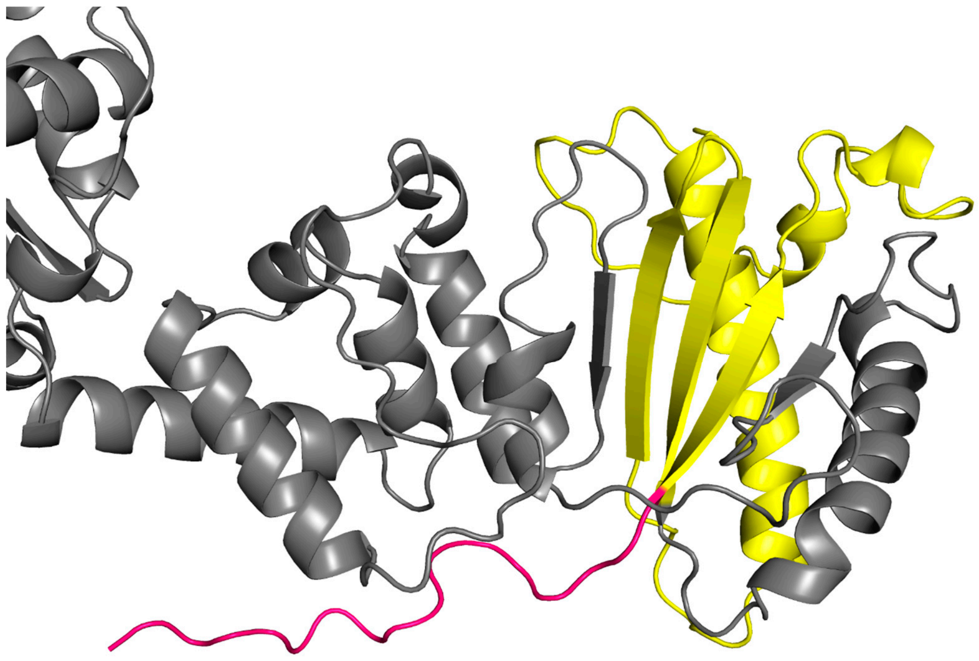

Figure S7. U94 3D model. MI108 protein portion is highlighted in yellow and the N-tail (aa 1-17) in fuchsia.

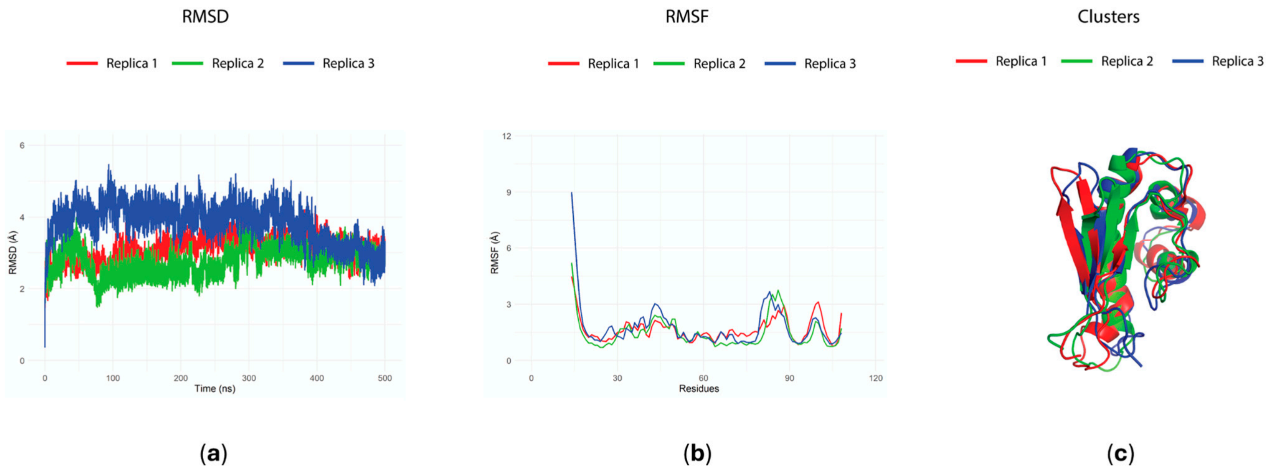

Figure S8. Trajectory analyses of the MD replicas of the KI95 fragment. (**a**) The RMSD of the backbone; (**b**) The RMSF of the backbone and (**c**) The superimposition of the most representative conformation of each replica.
